# Epidemiological and clinical characteristics of children with pertussis: a 6-year retrospective cohort study in Chengdu, China

**DOI:** 10.3389/fped.2025.1455345

**Published:** 2025-08-26

**Authors:** Qing Yang, Dong-Mei Wang, Qi An, Liang Huang

**Affiliations:** Department of Science and Education Division, Public Health Clinical Center of Chengdu, Chengdu, Sichuan, China

**Keywords:** pertussis, clinical characteristic, epidemiology, length of hospital stay, children

## Abstract

**Objective:**

This study aimed to analyze the epidemiological characteristics and clinical features of hospitalized children with pertussis who were admitted to the Chengdu Public Health Clinical Center (CPHCC), China from 2018 to 2023.

**Methods:**

We conducted a retrospective, anonymized study in children who were diagnosed with pertussis from January 2018 to December 2023 at the CPHCC. Univariate and multivariate Cox regression and the autoregressive integrated moving average model were used for identifying risk factors, and epidemiological trend analysis was performed.

**Results:**

We analyzed the data of 643 children with pertussis, 351 (54.59%) were younger than 6 months and 344 (53.50%) had a positive contact history. The mean (standard deviation) length of hospital stay was 8.6 (2.8) days. The median (interquartile range) number of days of coughing to the hospitalization time was 15 (12–20). In these patients, the most common clinical manifestations included a cough in 643 (100%), pneumonia in 364 (56.61%), paroxysmal spasmodic cough in 193 (30.02%), pulmonary rales in 175 (27.22%), and hoarseness of voice in 145 (22.55%). The most common complications were cardiomyopathy in 295 (45.88%) patients, liver damage in 255 (39.66%), and bronchopneumonia in 178 (27.68%). Univariate analysis and multivariate Cox proportional hazard model analysis suggested that an older age was beneficial for discharge from hospital (*P* < 0.05). However, patients who were treated with methylprednisolone and sulfamethoxazole had a longer length of hospital stay (both *P* < 0.05). An older age of onset, heavier weight, and therapeutic use of azithromycin contributed to a shorter length of hospital stay (all *P* < 0.05). A time series analysis showed a stable and predictable increase in pertussis cases.

**Conclusions:**

Heightened clinical and public health focus is needed for children with pertussis, particularly infants younger than 6 months. Public health investments should be increased, and specific measures should be developed to monitor and standardize the management of pertussis through multiple channels to interrupt the large-scale spread of this disease. Clinically, methylprednisolone is not recommended for treating pertussis.

## Introduction

Pertussis, known as whooping cough, with an estimated basic reproduction rate of 17 (1), is a highly contagious respiratory disease caused by *Bordetella pertussis*. Since the 1940s, the widespread use of whole-cell pertussis vaccine (DTwP) in combination with tetanus and diphtheria toxoids has dramatically reduced the incidence of pertussis (2). In China, the morbidity and mortality of pertussis have greatly declined since the implementation of the National Immunization Program in 1978, which was a public health success. In 2007, China expanded the coverage of vaccines in the National Immunization Program. The combined diphtheria-tetanus-acellular pertussis (DTaP) vaccine was introduced into the National Immunization Program. The DTaP vaccine had completely replaced the whole-cell pertussis vaccine by 2013 (3). Although vaccination coverage has been maintained at a high level, the incidence of pertussis has been reported to increase again after maintaining a low level for many years since the 1980s in developed countries. Furthermore, some areas have shown an increase in the incidence of pertussis every 3–4 years (4, 5).”

Children who have not completed full immunization, as well as adolescents and adults who have attenuated antibodies, are generally susceptible to pertussis. According to official data from China's National Bureau of Statistics (http://www.stats.gov.cn/), the incidence of pertussis in China has shown a fluctuating upward trend in recent years, especially since 2014. The morbidity rate of pertussis rose from 0.25/100,000 in 2014 to 35.10/100,000 in 2024 (6–8). The number of pertussis cases in 2024 (*n* = 494,321) was 2.91 times higher than the total number of cases from 2014 to 2023 (*n* = 169,738). In addition, the number of pertussis deaths in 2024 (*n* = 31) was 1.55 times higher compared to the total number of deaths from the disease in the past 10 years (*n* = 20) (unpublished data) (8).

According to previous studies, the reasons for the re-emergence of pertussis may involve switching the vaccine from whole-cell pertussis to DTaP, an antigen shift of *B. pertussis*, improvement of awareness and diagnostic capabilities, and declining vaccine efficacy and waning immunity (9). According to the Law of the People's Republic of China on Prevention and Control of Infectious Diseases, pertussis is classified as a Category B infectious disease. Additionally, the underdiagnosis and misdiagnosis of this disease undoubtedly increases the risk of it spreading in the community. Infants are particularly vulnerable to pertussis infection, especially those who have not completed basic immunization. Infants bear the greatest disease burden of severe cases and deaths (10, 11). With the increasing morbidity and mortality rates of pertussis, the pertussis outbreak is currently a public health problem that threatens human health in China and worldwide, and it requires urgent attention. Chengdu City, which is located in southwest China, is the provincial capital of Sichuan. The Chengdu Public Health Clinical Center (CPHCC), which is the only tertiary infectious disease hospital, is dedicated to infectious disease control and treatment. To some extent, data from the CPHCC reflect the epidemiology of pertussis in western China. We conducted this retrospective study to analyze the epidemiological characteristics, clinical features, and potential factors associated with the length of stay (LOS) in hospitalized children with pertussis in Chengdu, China from 2018 to 2023. To the best of our knowledge, there have been no reports of a large number of pertussis cases in this area to date in the relevant literature.

## Methods

### Study design

We retrospectively collected data from patients with pertussis who were admitted to the CPHCC from January 2018 to December 2023. Inclusion was based on the admission diagnosis and a positive nucleic acid test for *Bordetella pertussis*. The samples were analyzed for *Bordetella pertussis* nucleic acid using a PCR-fluorescent probe detection kit (DAAN GENE, Guangzhou, China), strictly adhering to the manufacturer's instructions. All of the hospitalized children with pertussis in this study were from Chengdu and surrounding areas. All available information, such as demographics, clinical manifestations, laboratory tests, and treatment and a history of diseases, on these cases were from the Hospital Information System.

### Criteria for the patients’ enrollment

The diagnosis of pertussis in children was based on the Diagnostic Criteria for Pertussis (WS 274-2007) from the Ministry of Health of the People's Republic of China, the Recommendations for the Diagnosis and Treatment of Pertussis in Children in China (Infectious Diseases Group of the Pediatrics Branch of the Chinese Medical Association, 2017), and the Expert Consensus of the Chinese Pertussis Action Plan (Chinese Preventive Medical Association, 2021).

### Statistical analysis

Statistical analyses were performed using STATA/MP 14.1 software (StataCorp, College Station, TX, USA) and R version 4.4.0 (R Foundation for Statistical Computing, Vienna, Austria). The SEAST module for STATA was installed to implement the Edward's seasonality test. *P* values <0.05 were considered statistically significant. To forecast the time series of case numbers, the autoregressive integrated moving average (ARIMA) model was applied by R forecast package version 8.22.0 with the build-in auto.fit() function for best fitting. Normally distributed continuous data are shown as the mean and standard deviation, and non-normally distributed continuous data are shown as the median and interquartile range (IQR). Since pertussis generally has a favorable prognosis, the length of stay (LOS) can serve as a sensitive indicator of disease severity. To identify factors influencing the disease course, we treated LOS as the time-to-event variable, defining “discharge” as the event of interest. Cox proportional hazards model with stepwise variable selection was applied to analyze the data. The intensity of the seasonal occurrence of pertussis was used by using Edward's analysis. We used a univariate analysis to examine the factors associated with discharge of children from the hospital. A multivariate Cox proportional hazard model analysis was then used to identify high-risk factors affecting discharge, which indicated the disease severity. The log-rank test was used to assess differences in the area under the curve of survival. The *χ*² or Fisher's exact test was used to compare and examine the differences between case characterization.

### Ethical approval and consent for clinical data use

This retrospective, noninterventional, anonymized study was approved by the Ethics Committee of Chengdu Public Health Clinical Center of Chengdu (YJ-K2023-55-01). Written consent for clinical data use was obtained from patients (>8 years old) and their legal guardians on admission to the hospital.

## Results

### Epidemiology

**Figure 1:**
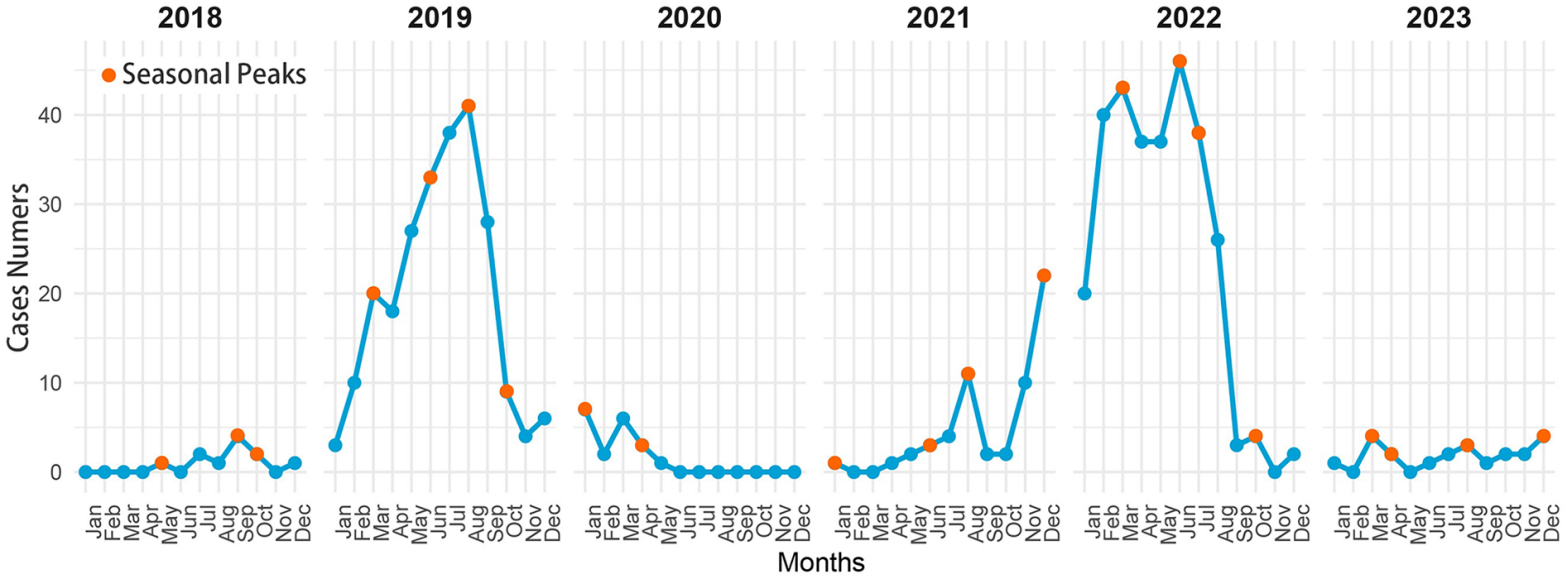
Time distribution of children hospitalized with pertussis at the CPHCC by month from 2018 to 2023.

Regarding the distribution of the hospitalization time, we observed an increase in the number of patients with pertussis starting in March with a peak in August, and these cases accounted for 70.14% (*n* = 451) of all cases in these 6 months. Moreover, the number of hospitalized cases of pertussis was highest in 2019 and 2022 (Figure 1). This finding indicated an increased incidence of pertussis in China. To predict trends in the progression of pertussis, we retrospectively collected information on pertussis outbreaks in Sichuan Province published by the Sichuan Center for Disease Control and Prevention from June 2016 to March 2024 (12). Edward's analysis suggested seasonality in the distribution of pertussis (*χ*² = 1279.15, *P* < 0.001).

**Figure 2:**
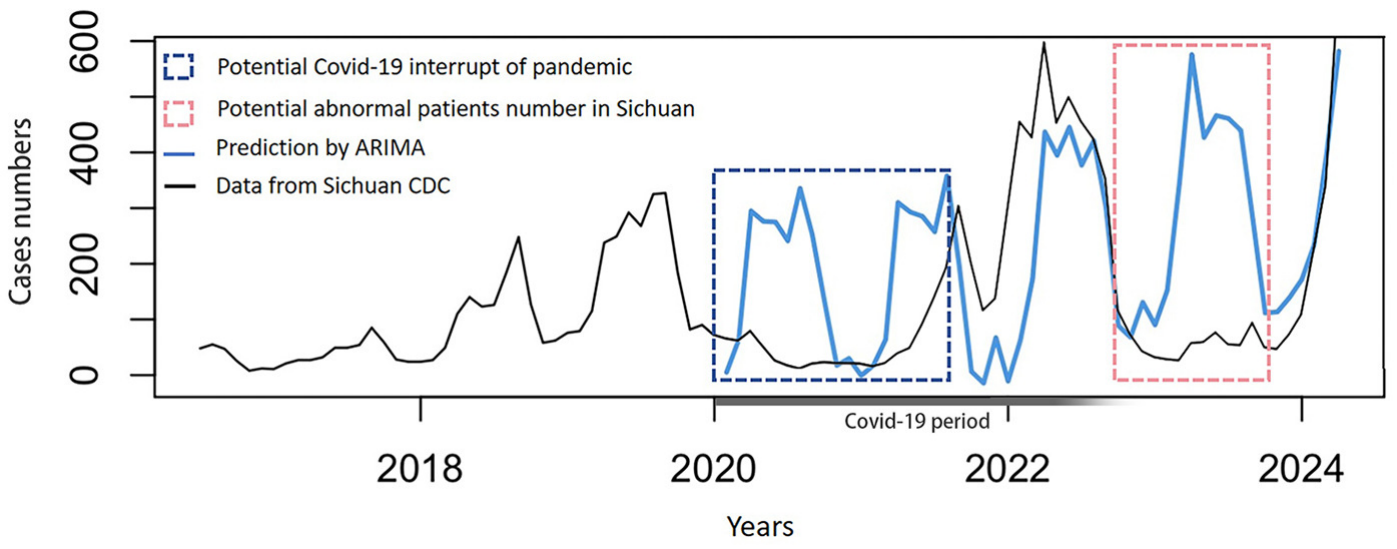
Prediction by the autoregressive integrated moving average model based on actual data published by the sichuan center for disease control and prevention on a monthly basis from June 2016 to march 2024.

We used the data published by the Sichuan Center for Disease Control and Prevention from June 2016 to December 2019 to train the ARIMA model, and then predicted the trend of the incidence of pertussis after 2020 [beginning of coronavirus disease 2019 (COVID-19) pandemic]. The forecast results suggested a significant difference between the predicted incidence of pertussis from 2020 to the end of 2021 and the actual occurrence, which was in the COVID-19 pandemic. The actual number of patients with pertussis in 2023 was substantially lower than the predicted number, but the prediction was consistent with the national epidemic trend (Figure 2).

### Basic characteristics of the patients

**Figure 3:**
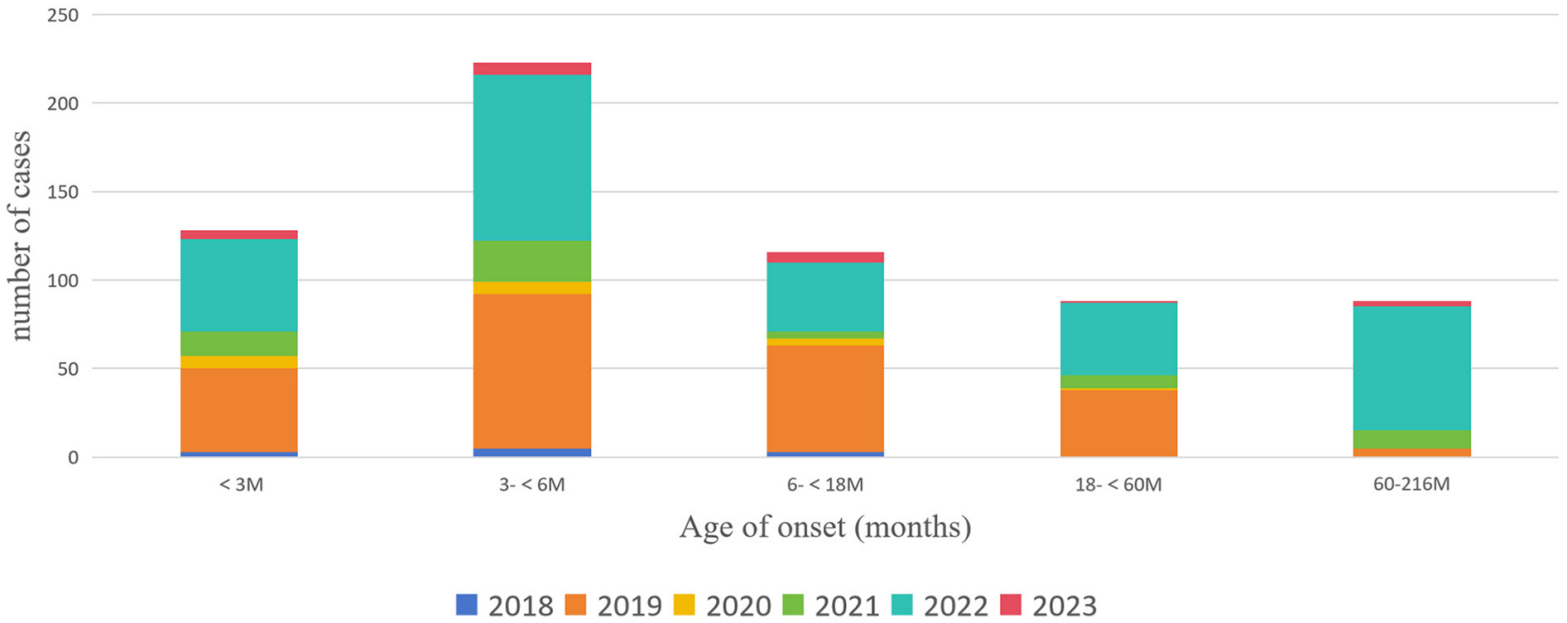
Distribution of the age of onset of children with pertussis who were hospitalized at the CPHCC from 2018 to 2023.

A total of 724 hospitalized children with pertussis were found. We excluded 32 duplicate hospitalizations, 45 automatic discharges, and 4 patients with combined congenital underlying diseases. The final cohort included 643 patients. A total of 47.43% (*n* = 305) of the patients were boys and 52.57% (*n* = 338) were girls. These patients were from Chengdu and its satellite cities. A total of 53.50% (*n* = 344) of the patients had a positive contact history. 19.91% (*n* = 128) of the children were younger than 3 months. These young children should not receive the first dose of DTaP vaccination according to the Chinese Immunization Program Vaccine Childhood Immunization Procedure (Figure 3).

The median age of the 643 pediatric patients was 5 months (IQR: 3–23 months) and the median weight was 8 kg (IQR: 6–11.5 kg). The patients’ median time from cough onset to hospitalization was 15 days (IQR: 12–20 days). Among the patients, the median C-reactive protein concentration (0.8 mg/L, IQR: 0.8–1.06 mg/L) and WBC (white blood cell) count (13.64, IQR: 9.83–19.59 × 10^9 ^/L) were above the normal limit at admission. Regarding the clinical presentation, all patients had cough symptoms (*n* = 643, 100%). Additionally, pneumonia (*n* = 364, 56.61%), paroxysmal rooster cough (*n* = 193, 30.02%), pulmonary rales (*n* = 175, 27.22%), hoarseness of voice (*n* = 145, 22.55%), and pharyngeal congestion (*n* = 109, 16.95%) were the next most common clinical manifestations. Fever, skin rash, and mental deficiency were also found. The most common complications were cardiomyopathy (*n* = 295, 45.88%), liver damage (*n* = 255, 39.66%), and bronchopneumonia (*n* = 178, 27.68%). The characteristics of children with pertussis hospitalized at the CPHCC from 2018 to 2023 are shown in Table 1.

**Table 1:** Characteristics of children with pertussis hospitalized at the CPHCC, 2018-2023.

Characteristics	*n* (%) Medians (IQR) Means (SD)
Demographic information	
Gender	
Male	305 (47.43)
Female	338 (52.57)
Age of onset (months)	5 (3-23)
Weight (Kg)	8 (6–11.50)
Positive contact history	
Yes	344 (53.50)
No	299 (46.50)
Cough to hospitalization time (days)	15 (12–20)
LOS, means ± SD, days	8.60 (2.80)
Age groups *n* (%)	
<3 M	128 (19.91)
3–5 M	223 (34.68)
6–17 M	116 (18.04)
18–59 M	88 (13.69)
60–216 M	88 (13.69)
Clinical manifestations *n* (%)	
Cough	643 (100.00)
Paroxysmal spasmodic cough	193 (30.02)
Fever	50 (7.78)
Hoarseness of voice	145 (22.55)
Pulmonary rales	175 (27.22)
Pharyngeal congestion	109 (16.95)
Skin rash	45 (7.00)
Pneumonia	364 (56.61)
Diarrhea	19 (2.96)
Apnea	10 (1.56)
Mental deficiency	17 (2.64)
Vomiting	14 (2.18)
Wheeze	7 (1.09)
Laboratory tests at admission	
WBCC	13.64 (9.83–19.59)
ANC	19.10 (10.60–30.90)
ALC	0.68 (0.57–0.76)
CRP	0.80 (0.80–1.06)
ALT	34 (23–52)
CK	69 (51–104)
CK-MB	27 (21–35)
HGB	119 (111–128)
PLT	420 (331–528)
Complications *n* (%)	
Cardiomyopathy	295 (45.88)
Liver damage	255 (39.66)
Pertussis encephalopathy	2 (0.31)
Anemia	21(3.27)
Bronchopneumonia	178(27.68)

IQR, interquartile range; SD, standard deviation; LOS, length of stay; M, months; WBCC, white blood cell count (*10^9^/L); ALC, absolute lymphocyte count (*10^9^/L); ANC, absolute neutrophil count (*10^9^/L); CRP, C-reactive protein (mg/dl); ALT, Alanine Aminotransferase (U/L); CK, Creatine Kinase (U/L); CK-MB, Creatine Kinase Isoenzymes (U/L); HGB, hemoglobin concentrations (g/L); PLT, platelet count (*10^9^/L).

### Univariate analysis

**Figure 4:**
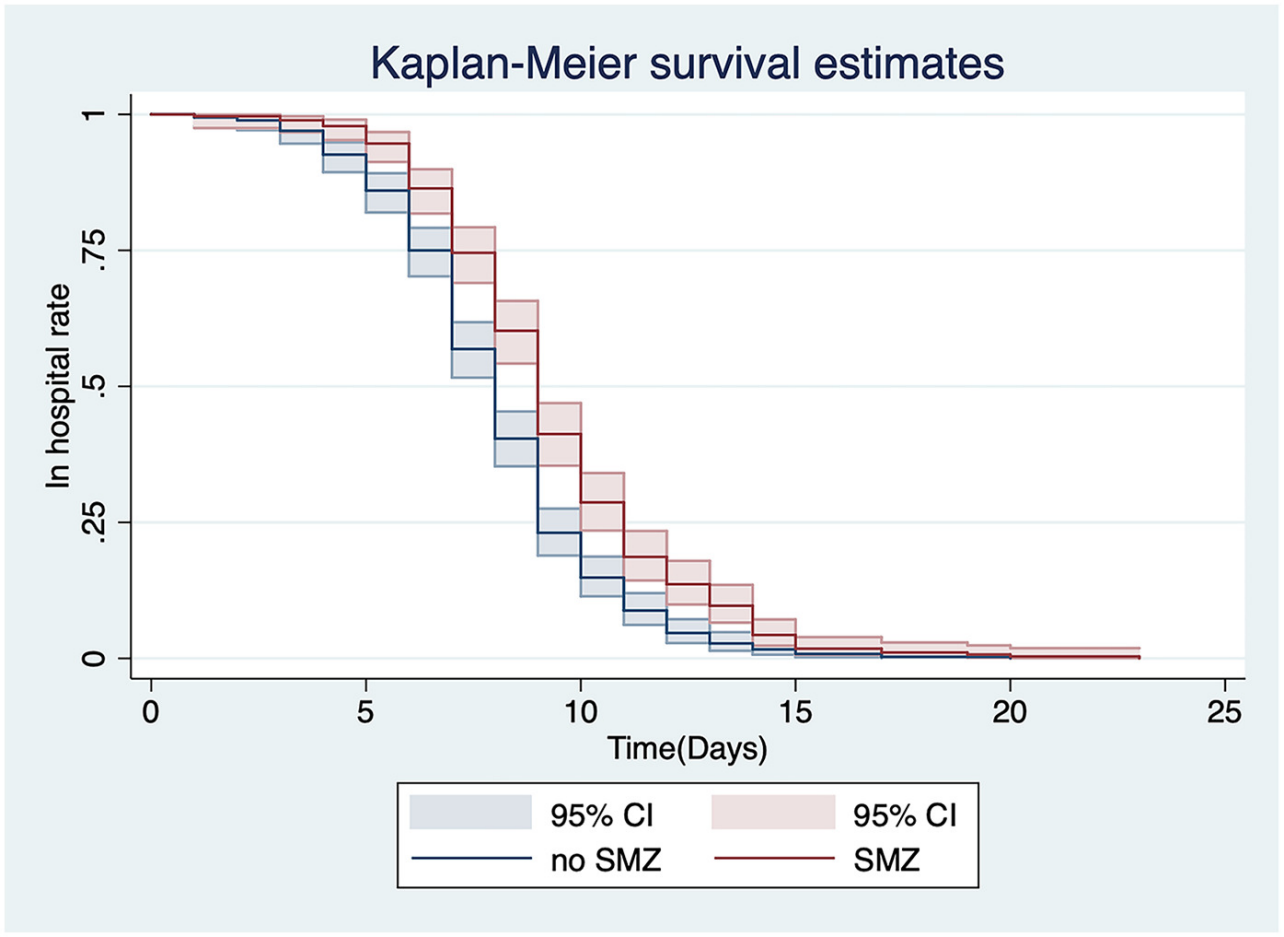
Univariate analysis of the relationship between the use of SMZ and discharge from the hospital.

**Figure 5:**
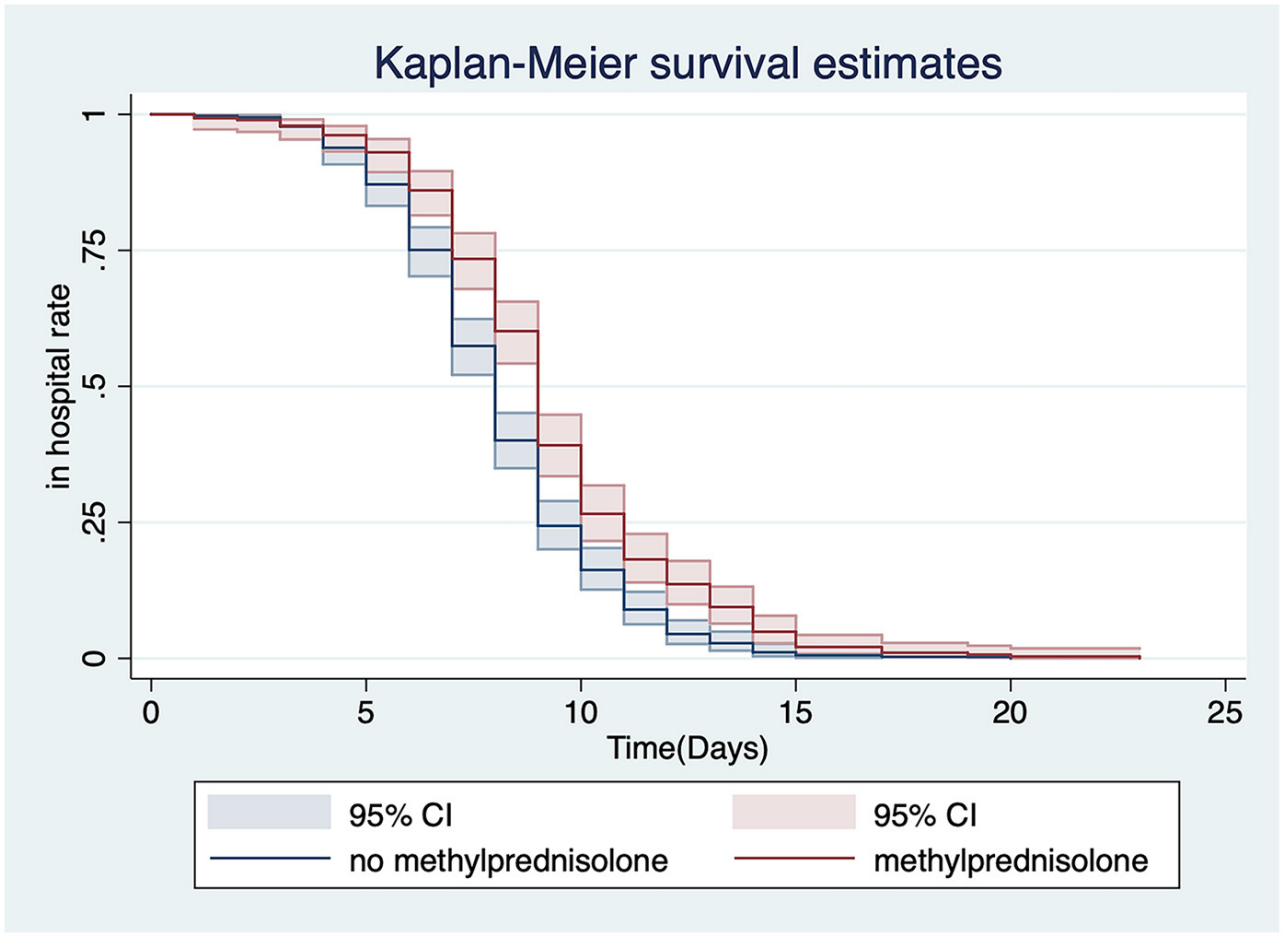
Univariate analysis of the relationship between the use of methylprednisolone and discharge from the hospital.

A univariate analysis was performed on all of the patients’ information, such as demographic data, clinical presentation, and treatment options (Table 2). We found that treatment with sulfamethoxazole (SMZ) [hazard ratio [HR] = 0.675[indicated 32.5% reduced discharge likelihood], 95% confidence interval [CI]: 0.577–0.791, *P* < 0.001] (Figure 4) and methylprednisolone (HR = 0.693, 95% CI: 0.592–0.811, *P* < 0.001) (Figure 5) affected the time to discharge of the children from the hospital, which was evidenced by prolonged hospital LOS (all *P* < 0.001). Furthermore, the children on older age (HR = 1.078, 95% CI: 1.016–1.143, *P* = 0.013) and treatment with azithromycin (HR = 1.186, 95% CI: 1.014–1.386, *P* = 0.033) had a shorter hospital LOS.

**Table 2:** Univariate analysis of risk factors affecting discharge in 643 hospitalized children with pertussis.

Variables	*n* (%)	HR	SE	Z	*P*-values	95% CI	
Age groups							
<3 M	128 (19.91)	1.078	0.032	2.48	0.013	1.016	1.143
3–5 M	223 (34.68)						
6–17 M	116 (18.04)						
18–59 M	88 (13.69)						
60–216 M	88 (13.69)						
Gender							
Male	305 (47.43)	1.027	0.082	0.34	0.733	0.879	1.200
Female	338 (52.57)						
Positive contact history	344 (53.50)	0.961	0.076	−0.5	0.618	0.822	1.123
Cough to hospitalization time (days)		1.002	0.003	0.59	0.558	0.996	1.008
Fever	50 (7.78)	0.977	0.144	−0.16	0.874	0.732	1.305
Pneumonia	364 (56.61)	0.871	0.069	−1.73	0.084	0.745	1.019
Azithromycin	284 (44.17)	1.186	0.095	2.13	0.033	1.014	1.386
SMZ	279 (43.39)	0.675	0.054	−4.87	<0.001	0.577	0.791
Cephalothin	84 (13.06)	0.889	0.104	−1	0.316	0.706	1.119
Methylprednisolone	286 (44.48)	0.693	0.056	−4.65	<0.001	0.592	0.811

HR, hazard ratio; SE, standard error; SMZ, sulfamethoxazole; M, months; CI, confidential interval.

### Patients who were grouped by age

We further examined the epidemiological information and clinical characteristics of hospitalized children with pertussis in Chengdu, China. All children enrolled in the study were divided into two groups: <6 months of age (not completed or just completed basic immunization, *n* = 351) and ≥6 months of age (*n* = 292) (Table 3). There were significant differences in sex, weight, positive contact history, and LOS between the two groups (all *P* < 0.05). Regarding clinical manifestations, the rates of pulmonary rales, pharyngeal congestion, fever, and pneumonia were significantly different between the two groups (all *P* < 0.05). Infants <6 months of age were more likely to develop pulmonary rales (*n* = 114, 32.48%) and pneumonia (*n* = 225, 64.10%) than those aged ≥6 months of age. The WBC count, WBC count ≥20 × 10^9 ^/L, neutrophil count, lymphocyte count, high neutrophil ratio (≥40%), high lymphocyte ratio (≥60%), creatine kinase isoenzymes, hemoglobin concentrations, and the platelet count were significantly different between the two groups (all *P* < 0.05) at admission. A WBC count ≥20 × 10^9 ^/L (*n* = 103, 29.35%) and a lymphocyte ratio ≥60% (*n* = 298, 84.90%) at admission were more common in children <6 months of age than in those ≥6 months of age. Regarding complications, cardiomyopathy (*n* = 182, 51.85%) and liver damage (*n* = 184, 52.42%) had higher incidences in children <6 months of age than in those ≥6 months of age, which suggested that these conditions were more severe in younger children.

**Table 3:** Demographic and clinical characteristics stratiﬁed by age.

Characteristics	Patients age <6 M (*n* = 351)	Patients age ≥6 M (*n* = 292)	*χ*²/Z	*P*-values
Demographic information				
Male	153 (43.59)	152 (52.05)	4.581	0.032*
Female	198 (56.41)	140 (47.95)		
Weight, median (IQR), Kg	6.3 (5.5–7)	12 (9–19)	20.401	0.0001*
Positive contact history	226 (64.39)	118 (40.41)	11.390	0.001*
Cough to hospitalization time, median (IQR), days	16 (12–21)	15 (12–20)	−0.464	0.643*
LOS, means ± SD, days	9 (7–10)	8 (6.5–10)	−3.520	<0.001*
Clinical manifestation				
Cough	351 (100.00)	292 (100.00)	/	1.000^a^
Paroxysmal spasmodic cough	114 (32.48)	79 (27.06)	2.232	0.135*
Fever	13 (3.70)	37 (12.67)	17.874	0.000*
Hoarseness of voice	88 (25.07)	57 (19.52)	2.812	0.094*
Pulmonary rales	114 (32.48)	61 (20.89)	10.806	0.001*
Pharyngeal congestion	48 (13.68)	61 (20.89)	5.894	0.015*
Skin rash	24 (6.84)	21 (7.19)	0.031	0.861*
Pneumonia	225 (64.10)	139 (47.60)	17.667	0.000*
Diarrhea	13 (3.70)	6 (2.06)	/	0.344^a^
Apnea	8 (2.28)	2 (0.69)	/	0.121^a^
Mental deficiency	8 (2.28)	9 (3.08)	/	0.348^a^
Vomiting	6 (1.71)	8 (2.74)	/	0.424^a^
Wheeze	4 (1.14)	3 (1.03)	/	0.600^a^
Laboratory tests at admission				
WBCC, median (IQR)	15.09 (10.68–21.32)	12.57 (8.97–16.97)	−4.236	0.000^b^
WBCC ≥ 20, *n* (%)	103 (29.35)	52 (17.81)	11.596	0.001*
ANC, median (IQR)	15.7 (9.4–23.4)	25.5 (15.45 −41.35)	8.131	0.000^b^
High NR (≥40%), *n* (%)	22 (6.27)	80 (27.40)	53.319	0.000*
ALC, median (IQR)	0.722 (0.641–0.78)	0.597 (0.466–0.70)	−9.991	0.000^b^
High LR (≥60%), *n* (%)	298 (84.90)	144 (49.32)	93.934	0.000*
ALT, median (IQR)	41 (30–63)	25.5 (19 −38)	−9.890	0.000^b^
CK, median (IQR)	68 (52–101)	73 (49–108)	−0.161	0.873^b^
CK-MB, median (IQR)	30 (24–39)	24 (19–31)	−7.304	0.000^b^
HGB, median (IQR)	117 (109–124)	123 (115–131.50)	5.998	0.000^b^
PLT, median (IQR)	473 (379–560)	366 (295–46)	−7.977	0.000^b^
Complications, *n* (%)				
Cardiomyopathy	182 (51.85)	113 (38.70)	11.106	0.001*
Liver damage	184 (52.42)	71 (24.30)	52.619	0.000*
Pertussis encephalopathy	2 (0.57)	0 (0.00)	/	0.503^a^
Anemia	6 (1.71)	15 (5.14)	5.927	0.015*
Bronchopneumonia	75(21.37)	103(35.27)	15.398	0.000*

M, months; IQR, interquartile range; LOS, length of stay; SD, standard deviation; WBCC, white blood cell count (*10^9 ^/L); ANC, absolute neutrophil count (*10^9 ^/L); NR, neutrophil ratio; ALC, absolute lymphocyte count (*10^9 ^/L); LR, lymphocyte ratio; ALT, alanine aminotransferase (U/L); CK, creatine kinase (U/L); CK-MB, creatine kinase isoenzymes(U/L); HGB, hemoglobin concentrations (g/L); PLT, platelet count (*10^9 ^/L);*P values were calculated by *χ*² test. ^a^*P* values were calculated by Fisher's exact test. ^b^*P* values were calculated by Mann–Whitney *U* test.

### Patients grouped by LOS in hospital

The hospital LOS has been reported to be an independent risk factor for severe pertussis and can objectively reflect the severity of this disease (13). The mean hospital LOS was 8.6 (standard deviation = 2.8) days. To further analyze the potential factors affecting the days of hospitalization of the pediatric patients, we divided the 643 children into two groups according to the mean LOS of 8.6 days. One group had an LOS ≤8 days (*n* = 328, 51.01%) and the other group had an LOS >8 days (*n* = 315, 48.99%). The factors that potentially affect the LOS were analyzed according to these groups (Table 4). We found significant differences in the age of onset (*χ*^2^ = 2.419, *P* = 0.016) and weight (*χ*^2^ = 2.231, *P* = 0.026) between the two groups. The age of onset and weight gain contributed to a shorter hospital LOS. The clinical manifestations and admission laboratory tests were compared between these two groups. We found that pulmonary rales, skin rash, the WBC count, alanine aminotransferase concentrations, and creatinine kinase concentrations were significantly different between the two groups (all *P* < 0.05). Regarding complications, cardiomyopathy (47.62% vs. 44.21%, *P* = 0.000) and liver damage (45.40% vs. 34.15%, *P* = 0.004) were significantly more frequent in children with an LOS >8 days than in those with an LOS ≤8 days. An analysis on antibiotic use showed that the percentage of children hospitalized for ≤8 days who were treated with azithromycin was higher than that of children hospitalized for >8 days (49.70% vs. 38.41%, *P* = 0.004). We also found that the proportion of children with pertussis treated with erythromycin, SMZ, or methylprednisolone was higher in children with an LOS >8 days than in those with an LOS ≤8 days (all *P* < 0.05).

**Table 4:** Demographic and clinical characteristics stratiﬁed by length of hospital stay.

Characteristics *n* (%) unless speciﬁed otherwise	LOS ≤ 8 days (*n* = 328)	LOS > 8 days (*n* = 315)	*χ*^2^/Z	*P*-values
Demographic informations				
Male	151 (46.04)	154 (48.89)	0.524	0.469*
Female	177 (53.56)	161 (51.11)		
Age of onset, median (IQR), months	6 (3–30.5)	4 (2–10.5)	2.419	0.016***
Weight median (IQR), Kg	8 (6.5–12.5)	4 (3–17)	2.231	0.026***
Positive contact history, *n* (%)	172 (52.44)	172 (54.60)	0.303	0.582*
Cough to hospitalization time, median (IQR), days	15 (12–20)	16 (12–20)	0.037	0.970***
Clinical manifestations, *n* (%)				
Cough	328 (100.00)	315 (100.00)	/	1.000**
Paroxysmal spasmodic cough	97 (29.57)	96 (30.48)	0.062	0.803*
Fever	22 (6.71)	28 (8.89)	1.066	0.302*
Hoarseness of voice	78 (23.78)	67 (21.27)	0.580	0.446*
Pulmonary rales	73 (22.26)	102 (32.38)	8.316	0.004*
Pharyngeal congestion	54 (16.46)	55 (17.46)	0.113	0.736*
Skin rash	30 (9.15)	15 (4.76)	4.746	0.029*
Pneumonia	174 (53.05)	190 (60.32)	3.456	0.063*
Diarrhea	10 (3.05)	9 (2.86)	0.021	0.886*
Apnea	5 (1.52)	5 (1.59)	/	1.000**
Mental deficiency	9 (2.74)	8 (2.54)	/	1.000**
Vomiting	7 (2.13)	7 (2.22)	/	1.000**
Wheeze	1 (0.31)	6 (1.91)	/	0.064**
Laboratory tests at admission				
WBCC, median (IQR)	13.06 (9.2–19.28)	14.68 (10.31–20.15)	−2.354	0.019***
WBCC ≥ 20 10^9 ^/L, *n* (%)	75 (22.87)	80 (25.40)	0.563	0.453*
ANC, median (IQR)	19.2 (10.6–30.8)	18.15 (10.7–31.95)	0.455	0.649***
High NR (≥40%), *n* (%)	50 (15.24)	52 (16.51)	0.192	0.661*
ALC, median (IQR)	0.68 (0.56–0.76)	0.71 (0.59–0.77)	−0.911	0.362***
High LR (≥60%), *n* (%)	217 (66.16)	225 (71.43)	2.077	0.150*
ALT, median (IQR)	31 (21–49)	36 (25–56)	−3.415	<0.001***
CK, median (IQR)	77 (55–110)	65 (47–93)	3.764	<0.001***
CK-MB, median (IQR)	27 (21–37.5)	27 (22–34)	−0.057	0.954***
HGB, median (IQR)	119 (111–128)	120 (111–127)	−0.538	0.591***
PLT, median (IQR)	408 (324–522)	436.5 (338–533)	−1.499	0.134***
Complications *n* (%)				
Cardiomyopathy	145 (44.21)	150 (47.62)	91.369	0.000*
Liver damage	112 (34.15)	143 (45.40)	8.499	0.004*
Pertussis encephalopathy	2 (0.61)	0 (0.00)	1.927	0.165*
Anemia	10 (3.05)	11 (3.50)	0.100	0.752*
Bronchopneumonia	102 (31.10)	76 (24.13)	3.9	0.048*
Treatments *n* (%)				
Erythromycin	91 (27.74)	117 (37.14)	6.486	0.011*
Azithromycin	163 (49.70)	121 (38.41)	8.294	0.004*
SMZ	111 (33.84)	168 (53.33)	24.854	0.000*
Cephalothin	43 (13.11)	41 (13.02)	0.001	0.972*
Quinolone	2 (0.61)	2(0.63)	/	1.000**
Methylprednisolone	114(34.76)	172(54.60)	25.630	0.000*

LOS, length of stay; IQR, interquartile range; WBCC, white blood cell count (*10^9 ^/L); ANC, absolute neutrophil count (*10^9 ^/L); NR, neutrophil ratio; ALC, absolute lymphocyte count (*10^9^/L); LR, lymphocyte ratio; ALT, Alanine Aminotransferase(U/L); CK, Creatine Kinase(U/L); CK-MB, Creatine Kinase Isoenzymes(U/L); HGB, hemoglobin concentrations (g/L); PLT, platelet count (*10^9 ^/L); SMZ, Sulfamethoxazole; *P values were calculated by *χ*² test. **P values were calculated by Fisher's exact test. ***P values were calculated by Mann–Whitney *U* test.

### Multivariate Cox proportional hazard model analysis

The multivariate Cox proportional hazard model analysis showed that an older age (HR = 1.093, 95% CI: 1.028–1.161, *P* = 0.004), methylprednisolone use (HR = 0.793, 95% CI:0.669–0.941, *P* = 0.008), and whether used SMZ alone (HR = 0.714, 95% CI: 0.600–0.848, *P* < 0.001) were independent risk factors affecting discharge of hospitalized children with pertussis (Table 5).

**Table 5:** Multivariate analysis of risk factors affecting discharge in 643 hospitalized children with pertussis.

Variables	*n* (%)	HR	SE	Z	*P*	95% CI	
Methylprednisolone	286 (44.48)	0.793	0.069	−2.65	0.008	0.669	0.941
Age groups		1.093	0.034	2.86	0.004	1.028	1.161
<3 M	128 (19.91)						
3–5 M	223 (34.68)						
6–17 M	116 (18.04)						
18–59 M	88 (13.69)						
60–216 M	88 (13.69)						
SMZ	279 (43.39)	0.714	0.063	−3.83	<0.001	0.600	0.848

HR, hazard ratio; SE, standard error; SMZ, sulfamethoxazole; M, months; CI, confidential interval.

## Discussion

### Stable increasing trend of pertussis cases

From 2018 to 2023, Sichuan Province has experienced a significant resurgence of pertussis, with a notable increase in cases observed in 2024. Data from the Sichuan Center for Disease Control and Prevention (2016–2024) show a stable upward trend, with peaks in 2019 and 2022, aligning with national trends where the incidence rate surged from 0.25/100,000 in 2014 to 35.10/100,000 in 2024 (6–8). In recent years, there has been an increase in pertussis cases in China and many other Asian countries (14, 15). Globally, Numerous studies have shown that, pertussis has shown persistent outbreaks in countries with a high vaccination coverage (16, 17). The data published by the Chinese Center for Disease Control and Prevention and China's National Bureau of Statistics have also suggested that the morbidity and mortality of pertussis have increased in recent years in China. Simultaneously, there is an urgent need for public health measures to protect susceptible populations from infections. The National Disease Control and Prevention Administration from China, in conjunction with the National Health Commission of the People's Republic of China, have developed the Pertussis Prevention and Control Program (2024 Edition). Which clarified the general requirements for prevention and control work, specific measures, requirements for case report management, multi-channel surveillance, vaccination, post-exposure prophylaxis, handling of aggregated outbreaks, prevention and control of outbreaks in key institutions, and laboratory testing (18). Additionally, the Pertussis Prevention and Control Program (2024 Edition) explicitly requires that there is a need to intensify target gene or whole gene sequencing of positive cultures of *B. pertussis*. Currently, the global pertussis epidemic has become a public health concern. While the morbidity and mortality rates of pertussis have been increasing in China for many years, the situation has further worsened since early 2024. Therefore, this study investigated the epidemiological and clinical characteristics of children with pertussis who were hospitalized at the CPHCC, and the factors affecting the hospital LOS were analyzed as an indicator of disease severity. This information could provide evidence to enhance the effective management of hospitalized patients with pertussis.

The ARIMA model, which had nearly perfect prediction in 2022, 2023 (compared with national data, not Sichuan data), and 2024, suggested stable and predictable increasing trends of pertussis. The discrepancy between observed and predicted pertussis cases in 2020 and 2021 is attributable to COVID-19 pandemic control measures. COVID-19 control strategies, such as social isolation and wearing of masks, substantially reduced the risk of pertussis. However, the trend of pertussis outbreaks immediately returned after restrictions due to COVID-19 were eased. The rapid resurgence of pertussis cases following the easing of COVID-19 restrictions heightened public health concerns in Sichuan, particularly with the surge in early 2024. These results suggest that urgent attention should be paid to reducing the trend of an increase in pertussis outbreaks.

### Seasonal pattern of outbreaks

In the present study, pertussis occurred throughout the year, from March to a peak in August. The data available from the Sichuan Center for Disease Control and Prevention and the CPHCC are consistent with those published by the UK Government and other studies (4, 19). These data suggest that pertussis is a cyclical disease that increases every 3–4 years, and usually peaks in the third quarter of the year. Using data published by the Chinese Center for Disease Control and Prevention and China's National Bureau of Statistics, we found that the morbidity rate of pertussis in China significantly declined from 2020 to 2021. However, this rate began to rapidly rise in 2022 and remained at a high level. The morbidity and mortality rates were even at multi-year highs in the first quarter of 2024. The seasonality of pertussis indicates that the public's awareness of pertussis should be strengthened. Control strategies should be implemented to minimize underreporting by multi-channel surveillance during the high-incidence seasons to minimize the spread of pertussis outbreaks. Whole genome sequencing studies of *B. pertussis* should also be conducted because it could help vaccine development and improvement.

### Younger patients have a higher risk of severity of pertussis

Physical precautions, such as keeping a social distance, wearing a mask and washing hands frequently lead to rare exposure to *B. pertussis*, but weaken the body's natural level of immunity to pertussis and decrease the level of antibodies passed from mothers to their newborns. With the relaxation of control measures, the morbidity of pertussis has rapidly increased (20). An insufficient level of immunity in the early stages of life in unvaccinated infants makes them more likely to be susceptible to pertussis infection. China's birth rate and natural population growth rate have continued to decline since 2017, but the incidence of pertussis remains high (21). This finding indicates that the actual prevalence of pertussis in China is even worse than originally believed.

In this study, cough, pneumonia, paroxysmal spasmodic cough, pulmonary rales, and hoarseness of voice were the most common clinical manifestations in hospitalized children. The top three complications were cardiomyopathy, liver damage, bronchopneumonia. Among the children included in our study, those younger than 6 months (54.59%) were at greater risk of hospitalization, which is consistent with the findings of previous studies (22, 23). This finding may be related to the fact that children at this stage are too young to have completed or just completed their basic immunizations. The subgroup analysis showed that pneumonia was more common in children <6 months of age. A younger age and longer hospital LOS were associated with a higher risk of cardiomyopathy and liver damage. These findings are consistent with a previous study that younger infants are more likely to experience complications (11). Our study showed that the time of cough onset to hospitalization time was 15 days, and up to 53.50% of children with pertussis had a confirmed history of positive cough exposure, including exposure to members of families, childcare facilities, and schools. The rate of underdiagnosing pertussis is high because of the low detection rates of pertussis in adults, coupled with nonspecific clinical symptoms that are not easily recognized in the early stages of this disease. Therefore, laboratory tests such as PCR testing of respiratory secretions need to be performed as soon as possible in patients with suspected pertussis for its early diagnosis and treatment to protect young infants. These tests are important in unvaccinated younger children because they are more likely to develop severe pertussis (24, 25). Protecting younger children from pertussis infection requires social and governmental attention and consideration. The World Health Organization has stated that pertussis vaccination should reduce the risk of severe pertussis in infants and young children, and recommends early and timely vaccination starting at 6 weeks and not later than 8 weeks of age (26). During the writing of this article, the Chinese government announced a new development. From January 1, 2025, China's immunization schedule for the DTaP vaccine was adjusted to include one dose each at 2, 4, 6, 18 months, and 6 years of age. The government was also emphasizing the importance of maintaining a consistently high level of vaccination coverage for DTaP (27).

Clinical studies in China report a high rate of erythromycin resistance among pertussis isolates, typically exceeding 70% (28). This resistance makes SMZ a potential alternative treatment option. However, specific data on the efficacy of SMZ against pertussis are still limited (29). The univariate analysis showed that discharge from hospital was faster if azithromycin used as an antibiotic. Additionally, this study suggested that the use of SMZ and methylprednisolone might be associated with a longer LOS. To further understand the relevant variables affecting the discharge of children from the hospital, a multivariate Cox proportional hazard model analysis was performed and showed that patients were more likely to be discharged sooner as their age increased. In this multivariate analysis, methylprednisolone and SMZ were independent risk factors that affected the discharge of hospitalized children with pertussis. The “Recommendations for the Diagnosis and Treatment of Pertussis in Chinese Children” (Infection Group, Chinese Medical Association Pediatrics, 2017) state that macrolide antibiotics should be used in patients with pertussis in all age groups. However, because of the high rate of macrolide resistance in *B. pertussis* in China, several studies have suggested that SMZ should be used to treat pertussis infected by macrolide-resistant strains (30, 31). Therefore, children who are clinically treated with SMZ are mostly those who have failed initial treatment with macrolides. The longer hospital LOS in children with pertussis treated with SMZ may be related to the progression of this disease owing to failure of children's drug-resistant primary treatment.

Our subgroup analysis of children according to the mean hospital LOS showed that an older age of onset and weight were associated with a shorter LOS. In children with a longer LOS, the proportions of children with pneumonia and pulmonary rales were higher than those in children with a shorter LOS. This difference was significant for pulmonary rales. The presence of pulmonary rales indicates the existence of lung disease. Regarding laboratory tests, the WBC count and alanine aminotransferase concentrations were significantly higher in children with a longer LOS than in those with a shorter LOS. The proportion of children using azithromycin was higher in those with a shorter LOS than in those with a longer LOS. However, the proportions of children using erythromycin, SMZ, and methylprednisolone were higher in those with a longer LOS than in those with a shorter LOS. Our findings suggest that methylprednisolone is not recommended for pertussis treatment. This recommendation is consistent with that described by Cherry regarding steroid use (32).

### Limitations

This study has some limitations. First, the statistical analysis was limited by the number of cases and the source of patients because this was a single-center study. Additionally, some details of symptoms may not have been recorded in the medical records, thus yielding results that may have been different in other regions or hospitals. Second, the effects of the COVID-19 pandemic on pertussis could not be avoided, as the study period included this time. Third, there were no follow-up data for the patients in the long term. Finally, this study found extremely high vaccination coverage among age-eligible children. Medical records with initially unclear vaccination status were later confirmed to be fully vaccinated as required, making it difficult to include them in this study for differential analysis.

## Conclusion

This study highlights the importance of public health interventions to curb the spread of pertussis amid its rising incidence. Physicians should monitor younger children with pertussis, especially infants <6 months of age, who are more susceptible to pneumonia, cardiomyopathy, and liver damage. Methylprednisolone is not recommended for general treatment of pertussis.

## Data Availability

The raw data supporting the conclusions of this article will be made available by the authors, without undue reservation.
